# High‐throughput sequencing reveals the core gut microbiome of Bar‐headed goose (*Anser indicus*) in different wintering areas in Tibet

**DOI:** 10.1002/mbo3.327

**Published:** 2016-02-04

**Authors:** Wen Wang, Jian Cao, Fang Yang, Xuelian Wang, Sisi Zheng, Kirill Sharshov, Laixing Li

**Affiliations:** ^1^ Key Laboratory of Adaptation and Evolution of Plateau BiotaNorthwest Institute of Plateau BiologyChinese Academy of SciencesXi'ning810000China; ^2^ Center of Growth, Metabolism and AgingCollege of Life SciencesSichuan UniversityChengdu610000China; ^3^ University of the Chinese Academy of SciencesBeijing100101,China; ^4^ Research Institute of Experimental and Clinical MedicineNovosibirsk630117Russia

**Keywords:** Artificial breeding, bar‐headed geese, gut microbiome, illumina high‐throughput sequencing, probiotics

## Abstract

Elucidating the spatial dynamic and core gut microbiome related to wild bar‐headed goose is of crucial importance for probiotics development that may meet the demands of bar‐headed goose artificial breeding industries and accelerate the domestication of this species. However, the core microbial communities in the wild bar‐headed geese remain totally unknown. Here, for the first time, we present a comprehensive survey of bar‐headed geese gut microbial communities by Illumina high‐throughput sequencing technology using nine individuals from three distinct wintering locations in Tibet. A total of 236,676 sequences were analyzed, and 607 OTUs were identified. We show that the gut microbial communities of bar‐headed geese have representatives of 14 phyla and are dominated by *Firmicutes*,* Proteobacteria*,* Actinobacteria*, and *Bacteroidetes*. The additive abundance of these four most dominant phyla was above 96% across all the samples. At the genus level, the sequences represented 150 genera. A set of 19 genera were present in all samples and considered as core gut microbiome. The top seven most abundant core genera were distributed in that four dominant phyla. Among them, four genera (*Lactococcus*,* Bacillus*,* Solibacillus*, and *Streptococcus*) belonged to *Firmicutes*, while for other three phyla, each containing one genus, such as *Proteobacteria* (genus *Pseudomonas*), *Actinobacteria* (genus *Arthrobacter*), and *Bacteroidetes* (genus *Bacteroides*). This broad survey represents the most in‐depth assessment, to date, of the gut microbes that associated with bar‐headed geese. These data create a baseline for future bar‐headed goose microbiology research, and make an original contribution to probiotics development for bar‐headed goose artificial breeding industries.

## Introduction

The bar‐headed goose (*Anser indicus*) is endemic to Asia, breeding in selected wetlands on the high plateaus of central Asia (a discontinuous range from Kyrgyzstan in the west to central China, and as far as Mongolia in the north) (Takekawa et al. 2009), wintering in the south‐central Tibet (more than 25% of the world populations) (Bishop et al. 1997) and India (about 50% of the world populations) (Javed et al. 2000). This goose species, with a total worldwide populations of 60,000, currently does not approach the thresholds for vulnerable and is evaluated as Least Concern under the new IUCN Red List criteria (version 3.1) (IUCN 2012). Bar‐headed geese migrate along the central Asian flyway, and up to half of the world's populations fly over the Himalayan Mountains on their biannual migration between central Asia and India (Hawkes et al. 2011). Therefore, bar‐headed geese provide an exceptional opportunity to understand the molecular and physiological bases of high‐altitude adaptation.

There is no scientific research about the changes in physiological and behavioral traits during the early domestication of wild bar‐headed geese. This lack of knowledge was due in part to the underdeveloped industry of bar‐headed goose artificial breeding. Artificial breeding of bar‐headed geese began in 2003 at Mozhugongka County, about 100 km east of Lhasa, Tibet, by the “Lhasa Nida Natural Ecology Development Co., Ltd.” (Feare et al. 2010). By now, small‐scaled and regional‐scattered bar‐headed geese artificial breeding industries have been established in several provinces of China to meet the market demands and to protect this species. The biggest problem of bar‐headed geese artificial breeding is the low egg‐laying rate, which limit the number of eggs for artificial incubation. Our previous comparative study on the different bar‐headed geese breeding patterns has shown that marked differences in gut microbiota existed between wild and farmed bar‐headed geese (Wang et al. 2016). Considering that gut microbiome may be a crucial factor in the regulation of reproduction, we try to figure out the core gut microbes of bar‐headed geese at a wide range of scales.

The role of the gut microbiome in health and disease has emerged as an area of major scientific and clinical importance in the past 10 years (Dave et al. 2012; Cénit et al. 2014). Gut microbial mutualisms, commensalisms, and pathogenic relationships play an important role in several fundamental and crucial processes such as host development (McFall‐Ngai et al. 2013), immune homeostasis (El Aidy et al. 2014), nutrient assimilation (Kau et al. 2011), vitamins synthesis, metabolizing bile acids, sterols in the host (O'Mahony et al. 2015), and diseases (e.g., obesity (Zhao 2013), diabetes (Qin et al. 2012), and cancer (Garrett 2015)) in humans and other animals. Given the acknowledged importance of gut microbiome in vertebrate nutrient and energy metabolism and reproduction (Comninos et al. 2014), it should be of great research and practical application values to analyze the core gut microbiota of wild bar‐headed geese (Kohl 2012).

Among avians, gut microbiome research mainly focused on commercially farmed species such as chicken (Sergeant et al. 2014), turkey (Lu and Domingo 2008) and ostrich (Matsui et al. 2010). Only limited data are available about the gut microbiome of the wild birds such as parrots (Waite et al. 2012), South American folivorous hoatzins (Godoy‐Vitorino et al. 2012), penguins (Dewar et al. 2013), and New World vultures (Roggenbuck et al. 2014). Prior to this study, virtually nothing was known about the composition and structure of the gut microbiota related to the wild bar‐headed goose. In this article, 16S rRNA gene analysis was used to identify gut microbial community composition, and the fecal samples derived from different wintering flocks of bar‐headed geese in Tibet were compared to characterize the core gut microbes. This study represents the first census of gut microbes identified in various flocks of bar‐headed geese, with the ultimate goal to develop probiotics and enhance reproductive rate of this domesticated bird.

## Materials and Methods

### Ethics statement

The experiment complied with the Animal Management Rule of the National Health and Family Planning Commission, People's Republic of China (documentation 55, 2001), and fecal samples collection were approved by the Animal Care and Use Committee of the Chinese Academy of Sciences.

### Fecal samples collection

All the field works were permitted by the Administration for Wild animal and plant protection and Nature Reserve, Tibet Provincial Department of Forestry. A total of nine fresh bar‐headed geese fecal samples were collected from individuals in three wintering flocks inhabiting different areas (separated by 50 km) in Tibet on 10–11 February 2015. Three fecal samples were collected from overwintering flock 1 (F1) in Chexiu country (29^°^10′31.8″N, 88^°^28′03.1″E), Sajia county, Xigaze city, Tibet. Three fecal samples were collected from overwintering flock 2 (F2) in Nierixiong country (29^°^19′24.6″N, 88^°^50′28.2″E), Xigaze city, Tibet. Three fecal samples were collected from overwintering flock 3 (F3) in Chabalang country (29^°^22′18.0″N, 90^°^47′31.1″E), Qushui county, Lhasa city, Tibet. All three locations are the typical wintering habitats for bar‐headed geese. Approximately 1 g of feces were collected from the inner part of fecal balls, avoiding collection of fecal materials that were touching the ground. All samples were placed in sterile containers and transported to the laboratory in a car‐carried refrigerator. In laboratory, fecal samples were kept frozen at −80^°^C until processing.

### DNA extraction, PCR amplification, and illumina MiSeq sequencing

Microbial DNA was extracted from fecal samples using the E.Z.N.A.^®^ stool DNA Kit (Omega Bio‐tek, Norcross, GA) according to manufacturer's protocol. The V4–V5 region of the bacteria 16S rRNA gene were amplified by PCR (95°C for 2 min, followed by 25 cycles at 95°C for 30 sec, 55°C for 30 sec, and 72°C for 30 sec and a final extension at 72°C for 5 min) using primers 515F 5′ ‐ barcode ‐ (GTGCCAGCMGCCGCGG)‐3′ and 907R 5′ ‐ (CCGTCAATTCMTTTRAGTTT)‐3′, where barcode is an eight‐base sequence unique to each sample. PCR reactions were performed in triplicate, 20 *μ*L mixture containing 4 *μ*L of 5× FastPfu Buffer, 2 *μ*L of 2.5 mmol/L dNTPs, 0.8 *μ*L of each primer (5 *μ*mol/L), 0.4 *μ*L of FastPfu Polymerase, and 10 ng of template DNA. Amplicons were extracted from 2% agarose gels and purified using the AxyPrep DNA Gel Extraction Kit (Axygen Biosciences, Union City, CA) according to the manufacturer's instructions and quantified using QuantiFluor^™^ ‐ ST (Promega, Madison, WI, USA). Purified amplicons were pooled in equimolar and paired‐end sequenced (2 × 300) on an Illumina MiSeq platform according to the standard protocol. The raw reads were deposited into the NCBI Sequence Read Archive (SRA) database (Accession Number: SRP057952).

### Bioinformatic analyses

The raw fastq files were demultiplexed based on the barcode and primer sequence with the following criteria: (1) exact barcode matching, (2) two nucleotide mismatch in primer matching, (3) reads containing ambiguous characters were removed. Then, PE reads for nine fecal samples were run through Trimmomatic (version 0.33) (Bolger et al. 2014) to remove low‐quality base pairs using these parameters [SLIDINGWINDOW: 50: 20 MINLEN: 50]. Trimmed reads were then further merged using FLASH program (version 1.2.8) (Magocˇ and Salzberg 2011) with the parameters [‐m 10 ‐x 0.2 ‐p 33 ‐r 300 ‐f 450 ‐s 150].

The 16S sequences were analyzed using a combination of software UPARSE (usearch version v8.0.1517, http://drive5.com/uparse/) (Edgar 2013), QIIME (version 1.8) (Kuczynski et al. 2012), and R (version 3.1.2). The demultiplexed reads were clustered at 97% sequence identity into operational taxonomic units (OTUs) using the UPARSE pipeline (http://drive5.com/usearch/manual/uparse_cmds.html). The OTU representative sequences were aligned against to the greengenes reference template set (http://greengenes.lbl.gov/Download/Sequence_Data/Fasta_data_files/core_set_aligned.fasta.imputed) based on PyNAST (version 1.2.1) (Caporaso et al. 2010). The phylogenetic tree was constructed using FastTree (version 2.1.3) (Price et al. 2010) with the filtered alignment. The Ribosomal Database Project (RDP) Classifier (version 2.2) (Wang et al. 2007) was employed for taxonomy assignment against Greengenes (version gg_13_8) (DeSantis et al. 2006) with confidence score ≥0.8. For the alpha‐diversity metrics, richness estimators (ACE and Chao1), diversity indices (Shannon and Simpson) were calculated by mothur (version 1.32.1) (Schloss et al. 2009) and Rarefaction plots were generated with iterations of 10 at each sampling depth 100 and increments of 100. For the beta‐diversity metrics, the weighted UniFrac distance matrix (Lozupone and Knight 2005) were calculated and visualized with Principal Coordinate Analysis (PCoA) analyses in QIIME. Biomarker discovery analysis of each taxonomic unit was performed using LeFse (version 1.0.7) (Segata et al. 2011). All figures were generated with customized R scripts.

## Results

### Data summary

After filtering the low‐quality reads, trimming the longer homopolymer runs, adapters, barcodes and primers, and rarefying the datasets, 21,167 to 34,340 effective sequences were collected from each fecal sample, resulting in a total of 236,676 sequences from the nine samples. The total number of reads, number of base pairs, and the mean length of the reads obtained from the original fastq file of each fecal sample before and after quality control filters are presented in Table S1.

All the sequences were delineated into OTUs with 97% sequence similarity threshold, consistent with the other studies using deep sequencing methods (Waite et al. 2014). A total of 607 OTUs were obtained and each sample contained 130 to 389 OTUs (Table 1). For each OTU, the relative abundance was plotted for each sample in which the OTU was present. The most abundant OTUs tended to be present in more fecal samples than the less abundant OTUs overall (Fig. S1A). The top 20 most abundant OTUs in each sample were shown in Figure S1B. The Good's coverage ranged from 99.7 to 99.9%, indicating that the majority of bacterial phylotypes present in each sample were identified in this study. The number of OTUs covered 77.53–93.99% and 78.74–92.99% of the richness estimated by the ACE and Chao1 indices, respectively (Table 1).

**Table 1:** Number of operational taxonomic units (OTUs), estimated OTU richness (ACE and Chao1), diversity index (Shannon and Simpson) for each sample

Sample ID	OTUs	ACE	Chao1	Shannon	Simpson	Coverage (%)
F1_1	187	241.21	237.50	2.24	0.26	99.8
F1_2	208	252.09	256.22	2.34	0.23	99.8
F1_3	247	282.29	291.00	3.79	0.06	99.7
F2_1	282	335.64	348.05	4.12	0.03	99.7
F2_2	373	420.74	421.12	3.27	0.13	99.8
F2_3	389	413.89	418.33	4.45	0.04	99.8
F3_1	202	253.92	250.13	2.45	0.21	99.7
F3_2	258	302.08	304.00	2.90	0.18	99.7
F3_3	130	157.41	158.79	2.30	0.22	99.9

The observed species rarefaction curves were calculated for each sample to assess whether the depth of sequencing were large enough to yield a stable estimate of the species richness at the 97% similarity threshold. We found that the rarefaction curves reached the saturation plateau (Fig. S2A), indicating that sequencing depth was large enough to yield stable and unbiased estimates of species richness. We also used a species accumulation curve to determine if the bacterial diversity observed in our nine fecal samples represented the overall bacterial diversity present in bar‐headed geese gastrointestinal tract. This approach measures how many new OTUs are identified as additional samples are cumulatively added to the analysis. As shown in Figure S2B, the number of OTUs increased quickly at the range of 1 to 6 samples, and began to plateau by the end of our sampling, which indicated that we have largely saturated the bacterial diversity found in this situation.

### Composition of microbial community

Several different patterns of gut microbial compositions were distinguished in the comparison at each taxonomic level. Table S2 showed the number of taxonomic units detected in each samples. The compositions of microbial community at the levels of Class, Order, and Family were shown in Figure S3–S5.

**Figure 1:**
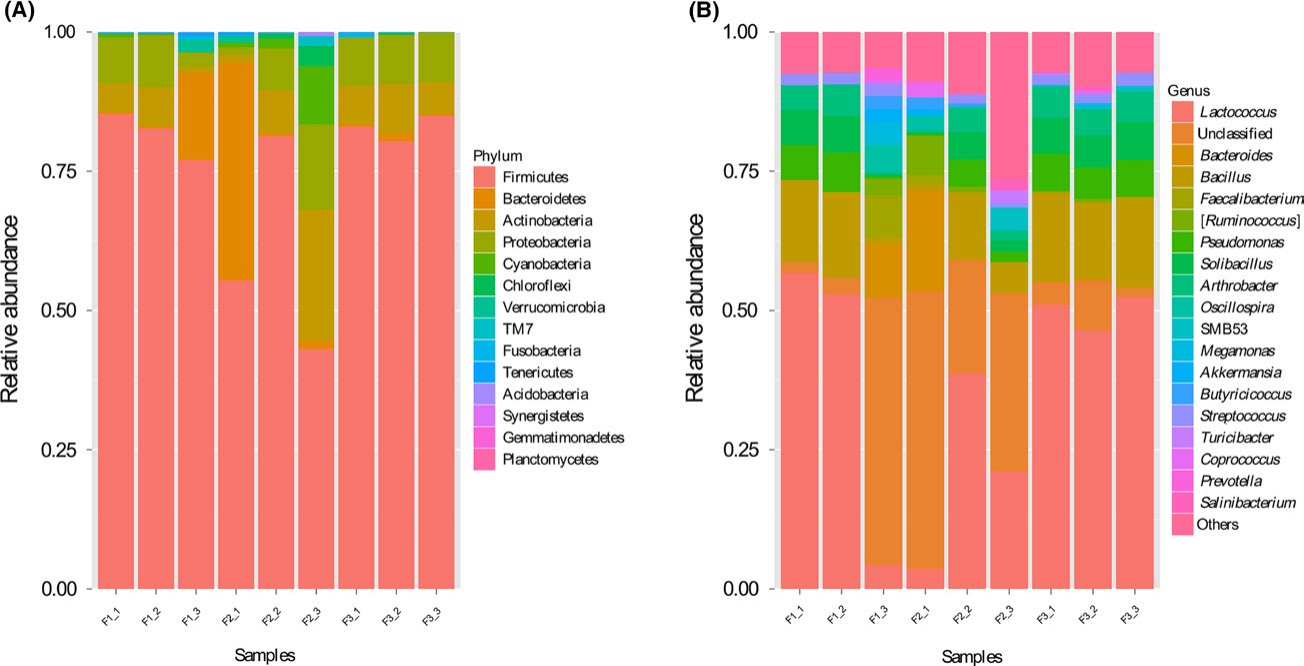
Characterization of the gut microbial community composition of each sample at the phylum and genus level.

The classification of sequences from the samples resulted in 14 different phyla that were identified in this study (Fig. 1A, Table S3). *Firmicutes* held the overwhelming predominance, with the average relative abundance of 74.78%, followed by *Proteobacteria* (7.84%), *Actinobacteria* (7.49%), and *Bacteroidetes* (6.65%). The additive abundance of these four most dominant phyla was above 96% across all the samples. *Chloroflexi*,* TM7* were the other two phyla that occurred in all the samples and the additive abundance accounted for 1%. Another eight phyla were only detected in one or several samples, including *Cyanobacteria*,* Verrucomicrobia*,* Tenericutes*,* Fusobacteria*,* Acidobacteria*,* Synergistetes*,* Planctomycetes,* and *Gemmatimonadetes*.

At the genus level, the sequences from the samples represented 150 genera (all genera named in Table S4). The top 20 genera were listed in Figure 1B. The sequences that could not be classified into any known genus were assigned as “unclassified”. The proportions of these unclassified genera varied between 1.70 and 49.91% among the different samples. These sequences represented 16.55% of the full dataset. *Lactococcus* was the most predominant genus, with relative abundance ranging from 4.27 to 56.67%. Other dominant genera in each samples were listed in Table S5. For each dominant genus, its distribution among the nine samples was either varied or consistent. For each sample, percentages of these genera were highly diversified.

### The gut microbiome relationships among samples

**Figure 2:**
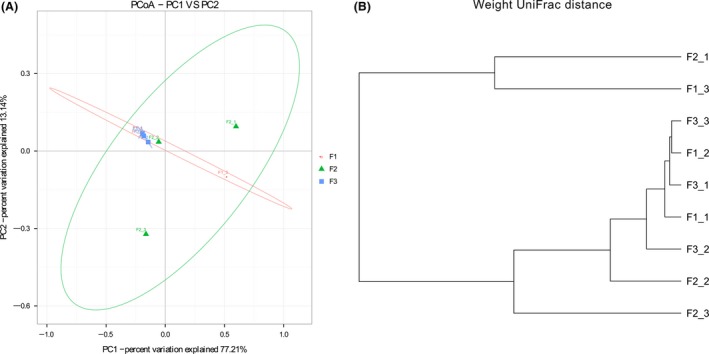
Principal Coordinate Analysis and hierarchical dendrogram plot of samples using the weighted UniFrac distance metric.

To survey the relationships of the gut microbial communities among nine samples, the weighted UniFrac PCoA and hierarchical dendrogram were analyzed (Fig. 2). There were obvious separated and overlapped samples for each of the wintering flocks. The fecal samples derived from F3 grouped closer than other two flocks. Samples F2_1, F2_3, and F1_3 exhibited higher changes in community compositions. These results confirmed that gut microbial communities of the majority of bar‐headed geese (6 of 9 samples) were significantly more similar.

### The core gut microbes at the genus level

A major research interest of this study was to determine whether a core gut microbiota is shared among all or the vast majority of the samples. To identify the core genera, we selected three core thresholds (as described by (Otani et al. 2014)): (1) genera present in all nine samples (100% core), (2) genera present in at least eight of nine samples (88.9% core), and (3) genera present in at least seven of nine samples (77.9% core).

Nineteen of the 150 genus‐level taxa identified were present in all nine samples at the 100% core threshold (Table 2). These 19 core genera were distributed among five phyla, and 68.42% of these genera were in the *Firmicutes* and *Proteobacteria*, with the remaining being in *Bacteroidetes*,* Actinobacteria*, and *Enterobacteria*. The proportion present in this 100% core threshold out of the total number of genera for each sample (showed in Table S2) ranged from 16.24% (sample F2_2) to 28.36% (sample F3_3). In addition to occupying a substantial portion of genera identified, the core community comprised 20.12–96.73% of the total number of quality‐filtered reads (showed in Table S4). The top seven core genera (Table 2) were observed in the top 20 genera (showed in Fig. 1A) and contributed the most to community abundance.

**Table 2:** The relative abundance of core genera (100% core threshold) in each sample

Phylum	Genus	The relative abundance (%)									
		F1_1	F1_2	F1_3	F2_1	F2_2	F2_3	F3_1	F3_2	F3_3	Average
Firmicutes	Lactococcus	56.67	52.93	4.27	3.52	38.74	21.05	50.97	46.43	52.37	36.33
Firmicutes	Bacillus	14.68	15.38	1.05	0.88	11.92	5.53	16.00	13.76	16.21	10.60
Proteobacteria	Pseudomonas	6.27	7.10	0.32	0.26	4.85	1.83	6.73	5.70	6.62	4.41
Firmicutes	Solibacillus	6.37	6.62	0.41	0.33	4.88	2.06	6.42	5.67	6.83	4.40
Actinobacteria	Arthrobacter	4.38	5.58	0.37	0.34	4.30	1.74	5.55	4.68	5.45	3.60
Bacteroidetes	Bacteroides	0.01	0.02	9.62	18.25	0.06	0.02	0.08	0.33	0.00	3.15
Firmicutes	Streptococcus	1.99	2.03	2.05	0.13	1.56	0.83	1.84	1.57	2.30	1.59
Firmicutes	Lysinibacillus	1.31	1.40	0.10	0.09	1.11	0.46	1.55	1.53	1.56	1.01
Firmicutes	Carnobacterium	1.13	1.44	0.05	0.07	1.09	0.53	1.20	1.03	1.38	0.88
	SMB53	0.04	0.04	0.69	0.25	0.14	4.13	0.21	0.45	1.02	0.77
Proteobacteria	Psychrobacter	0.67	0.76	0.03	0.06	0.59	0.22	0.77	0.66	0.74	0.50
Firmicutes	Lactobacillus	0.92	0.33	0.01	0.02	0.31	0.47	0.37	0.51	0.48	0.38
Firmicutes	Brochothrix	0.45	0.55	0.01	0.01	0.37	0.14	0.42	0.45	0.43	0.32
Firmicutes	Leuconostoc	0.26	0.56	0.02	0.02	0.38	0.11	0.56	0.31	0.48	0.30
Enterobacteria	Escherichia	0.10	0.12	1.05	0.02	0.21	0.07	0.16	0.13	0.17	0.23
Proteobacteria	Enhydrobacter	0.29	0.32	0.02	0.02	0.18	0.09	0.32	0.29	0.33	0.21
Proteobacteria	Acinetobacter	0.13	0.16	0.02	0.01	0.21	0.05	0.19	0.21	0.21	0.13
Actinobacteria	Pseudonocardia	0.08	0.16	0.01	0.01	0.17	0.46	0.03	0.07	0.00	0.11
Bacteroidetes	Flavobacterium	0.13	0.14	0.01	0.01	0.12	0.11	0.12	0.07	0.13	0.09

Using a slightly less strict core criterion (present in at least eight samples, 88.9% core threshold), another 14 genera were identified (Table S6). Using the even less strict criterion (present in at least seven samples, 77.9% core threshold), extra 18 genera were identified (Table S6).

## Discussion

Bar‐headed geese are currently one of the most popular species for the rare birds breeding industries in China. Recent studies have revealed important contributions of gut microbiome to health and disease in animals (Lee and Hase 2014). Thus far, studies regarding the microbial community of bar‐headed geese have been relatively limited. To the best of our knowledge, this study was the first one that characterized the core gut microbiome of bar‐headed goose with deep sequencing methodology.

The PCoA score plot revealed clearly that majority of bar‐headed geese harbored the common gut microbial communities even though these samples collected from different wintering locations. This identity may be related to the strict feeding habits of this species, which ingest mainly leaves and stems from *Gramineae* and *Cyperaceae* plants, and legume seeds (Middleton 1992).

The analysis of composition of gut microbiota demonstrated that the dominant bacteria of the nine samples belonged to four phyla, *Firmicutes*,* Proteobacteria*,* Actinobacteria*, and *Bacteroidetes*, which are commonly founded in the vertebrate gastrointestinal tract (Deng and Swanson 2015). These results are consistent with earlier studies on the gut bacterial assemblages of both adults and chicks in a wild population of black‐legged kittiwakes (*Rissa tridactyla*) (van Dongen et al. 2013). Our results are also consistent with a study on the cloacal microbiota of wild and captive parrots (Xenoulis et al. 2010). We further found that the top seven most abundant core genera (at 100% core threshold) were distributed in the four dominant phyla. Among them, four genera (*Lactococcus*,* Bacillus*,* Solibacillus*,* Streptococcus*) belonged to *Firmicutes*, while for other three phyla, each containing one genus, such as *Proteobacteria* (genus *Pseudomonas*), *Actinobacteria* (genus *Arthrobacter*), and *Bacteroidetes* (genus *Bacteroides*). We speculate that these predominant phyla and genera presented in the birds’ gut might potentially implicated in a variety of birds’ physiological processes (e.g., dietary carbohydrate, protein, DNA, and vitamin metabolism; immune homeostasis; and development). The different environment in this study did not result in marked difference, suggesting that some endogenous factors outweighed by far the environmental factors to shape the wild bar‐headed geese gut microbiota.

It should be of great research and practical application values to analyze these core gut microbes for probiotics development that may meet the demands of bar‐headed goose artificial breeding industries and accelerate the domestication of this species. The four representative dominant core genera belonged to each phylum were characterized through the literature and their predicted functions toward the host, as well as potential applications in the probiotics development and future bar‐headed goose microbiology research assessed.

*Lactococcus*, a genus of lactic acid bacteria from the family *Streptococcaceae*, are indigenous to food‐related habitats as well as associated with the mucosal surfaces of animals. The central metabolic pathways of this genus have been extensively studied because of their relevance in the industrial use of some species (e.g., *L. lactis*). These bacteria can convert a large fraction of the hexose sugar substrate to pyruvate via glycolysis and then to lactate in the redox balance step (Price et al. 2012). This may give *Lactococcus* a particular relevance to bar‐headed geese dietary carbohydrate and feeding habits. Given that *Lactococcus* are the focus of intensive research within the field of carbohydrate catabolism and industrial fermentation processes (Mayo et al. 2010), we will isolate and characterize functional bacterial of this genus from fecal samples in the future work, and develop probiotics for the bar‐headed geese breeding industries.

The genus *Pseudomonas* currently contains about 144 species (Gomila et al. 2015), and is ubiquitous in waters and soils, being one of the most adaptable bacteria to various environments. Members of the *Pseudomonas* produce diverse secondary metabolites affecting other bacteria, fungi and protozoa, but are also equipped with the capacity to secrete different types of ribosomally encoded toxic peptides and proteins (Ghequire and De Mot 2014). *Pseudomonas* produces as many as 795 bioactive substances, including 610 antibiotics and 185 substances with bioactive properties other than antibiotic activity, and some of these play very important roles in the biological control of pathogenic plant bacteria and in bioremediation (Isnansetyo and Kamei 2009). *Pseudomonas* is also thought to be one of the nitrogen fixing bacteria for plants and soil (Rediers et al. 2003), and has been isolated from the guts of *Tetraponera* ants (van Borm et al. 2002). However, the definite evidence on its role in birds gut microbiology is lacking. Whether *Pseudomonas* found in the gut of bar‐headed geese are of the nitrogen‐recycling endosymbionts or as an antimicrobial agent participate in the establishment of immune balance are worthy of further investigation.

Bacteria of the genus *Arthrobacter* are ubiquitous in soil environments. The ubiquity of *Arthrobacter* strains is considered to be due to their nutritional versatility and their pronounced resistance to desiccation, long‐term starvation, and environmental stress (Niewerth et al. 2012). In the context of gut microbial communities in vertebrates, *Arthrobacter* spp. isolated from Namaqua rock mice (*Aethomys namaquensis*) fecal contents could utilize xylose and produce three main types of short‐chain fatty acids (acetic, propionic, and butyric acids) (Johnson et al. 2006). Accordingly, we inferred that the presence of *Arthrobacter* in bar‐headed geese gut may be mainly related to their living environment and nutrient metabolism. In addition, *Arthrobacter* species are also used as probiotics for aquaculture. For example, *Arthrobacter sp. CW9*, isolated from guts of white shrimp (*Penaeus vannamei*), has both probiotic and immune‐stimulatory properties (Xia et al. 2014). Therefore, more detailed data are required to judge the role of *Arthrobacter* in wild bar‐headed geese.

*Bacteroides* species have extensive machinery to utilize the complex polysaccharides present in the colon as a source of carbon and energy. In doing so, the fermentative end products released by *Bacteroides* also provide nutrition and other beneficial properties to the host (Comstock 2009). There are many open questions regarding the bar‐headed goose‐*Bacteroides* mutualism. And it will be interesting to determine which dietary fiber is utilized by various *Bacteroides* species.

## Conclusion

Our knowledge of the role of gut microbiome in avian hosts lags far behind our understanding of mammalian systems. For the first time, we defined the core gut microbiome of bar‐headed goose in different wintering areas in Tibet. Our study creates a baseline for future bar‐headed goose microbiology research, and make an original contribution to probiotics development for bar‐headed goose artificial breeding industries. A total of 236,676 sequences from the nine samples were analyzed, and 607 OTUs were identified. Several different patterns of gut microbial community composition were compared at each taxonomic level. The gut microbial communities of bar‐headed geese had representatives of 14 phyla and were dominated by *Firmicutes*,* Proteobacteria*,* Actinobacteria*, and *Bacteroidetes*. At the genus level, the sequences represented 150 genera. A set of 19 genera were present in all samples and considered as core gut microbiome. The core gut microbes shared by all the samples might be associated with key physiological characteristics of bar‐headed geese and should be candidates for future probiotics development. These described data, in combination with ongoing metagenomics studies, should contribute to the domestication of bar‐headed goose.

## Conflict of Interest

None declared.

## Supporting information


**Figure S1.** Relative abundance of OTUs.

**Figure S2.** Impact of sequencing depth and sampling numbers on bacterial phylotypes detection.

**Figure S3.** Composition of Microbial Community at Class level.

**Figure S4.** Composition of Microbial Community at Order level.

**Figure S5.** Composition of Microbial Community at Family level.

**Table S1.** Raw data before and after standard quality control (QC) filters.

**Table S2.** Number of bacterial taxonomic units.

**Table S3.** The taxonomic composition at the phylum level of the microbial communities in each sample.

**Table S4.** The distribution of the sequences belonged to different genera in each sample.

**Table S5.** The top 18 most abundant genera in each sample.

**Table S6.** The distribution of sequences belonged to the newly added genera at 88.9 and 77.9% core threshold.
