# Supplementary figures and images for: High‐throughput sequencing reveals the core gut microbiome of Bar‐headed goose (Anser indicus) in different wintering areas in Tibet
Source: Microbiologyopen. 2016 Feb 4;5(2):287–95. doi: 10.1002/mbo3.327 (PMC4831473; doi:10.1002/mbo3.327)

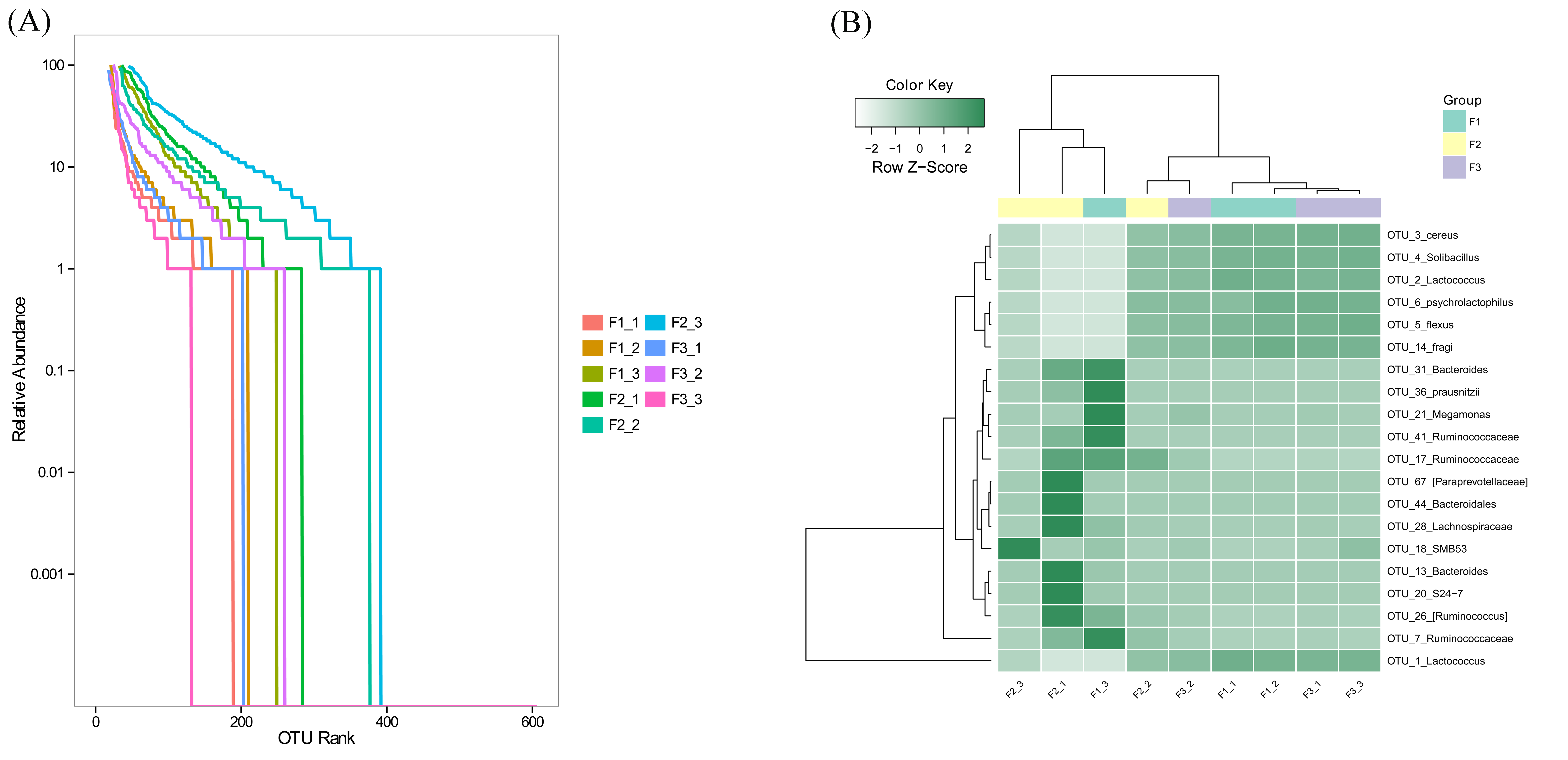

Supplement: Supplementary file 1 — Figure S1. Relative abundance of OTUs. [file MBO3-5-287-s001.tif]

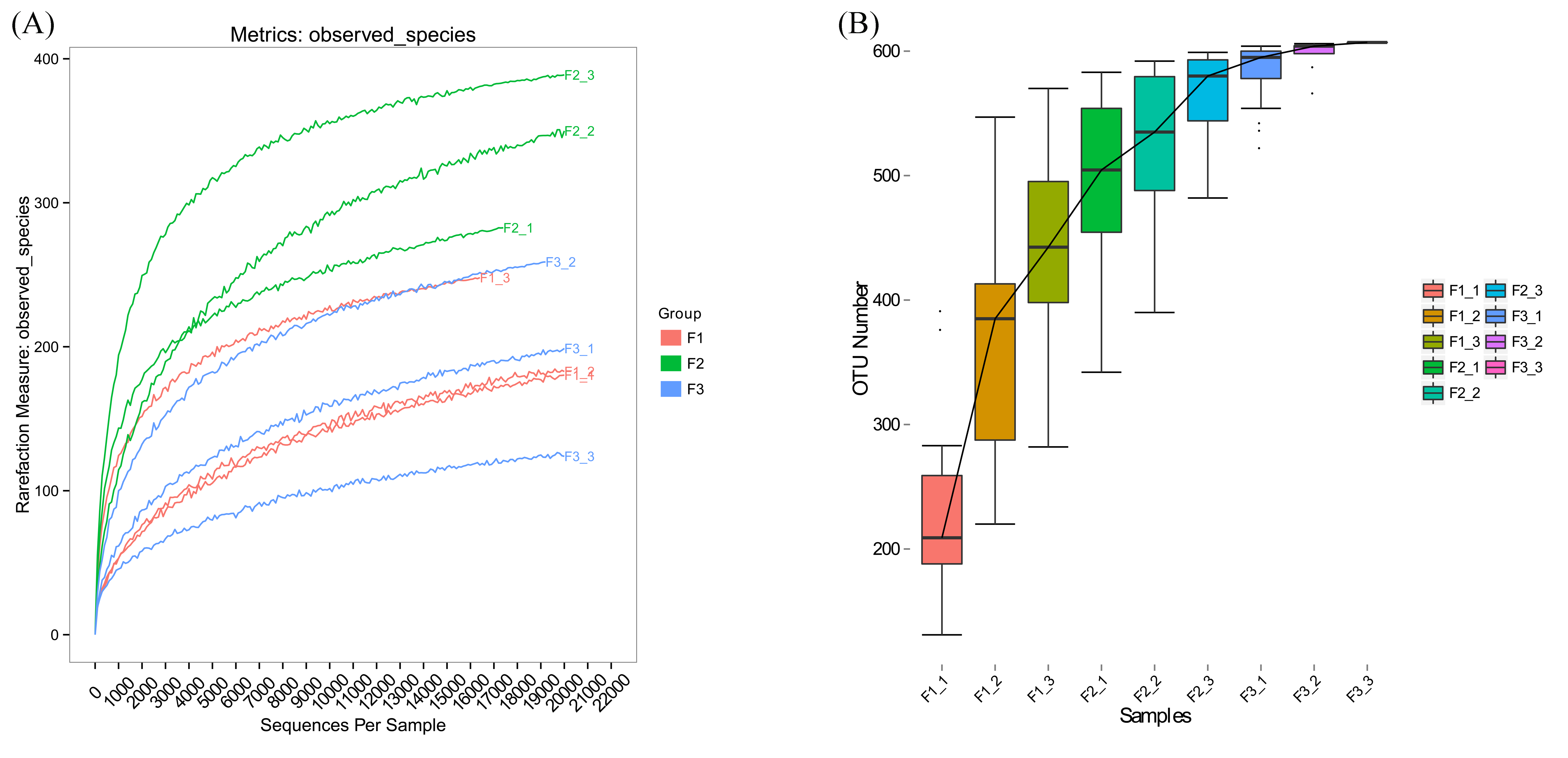

Supplement: Supplementary file 2 — Figure S2. Impact of sequencing depth and sampling numbers on bacterial phylotypes detection. [file MBO3-5-287-s002.tif]

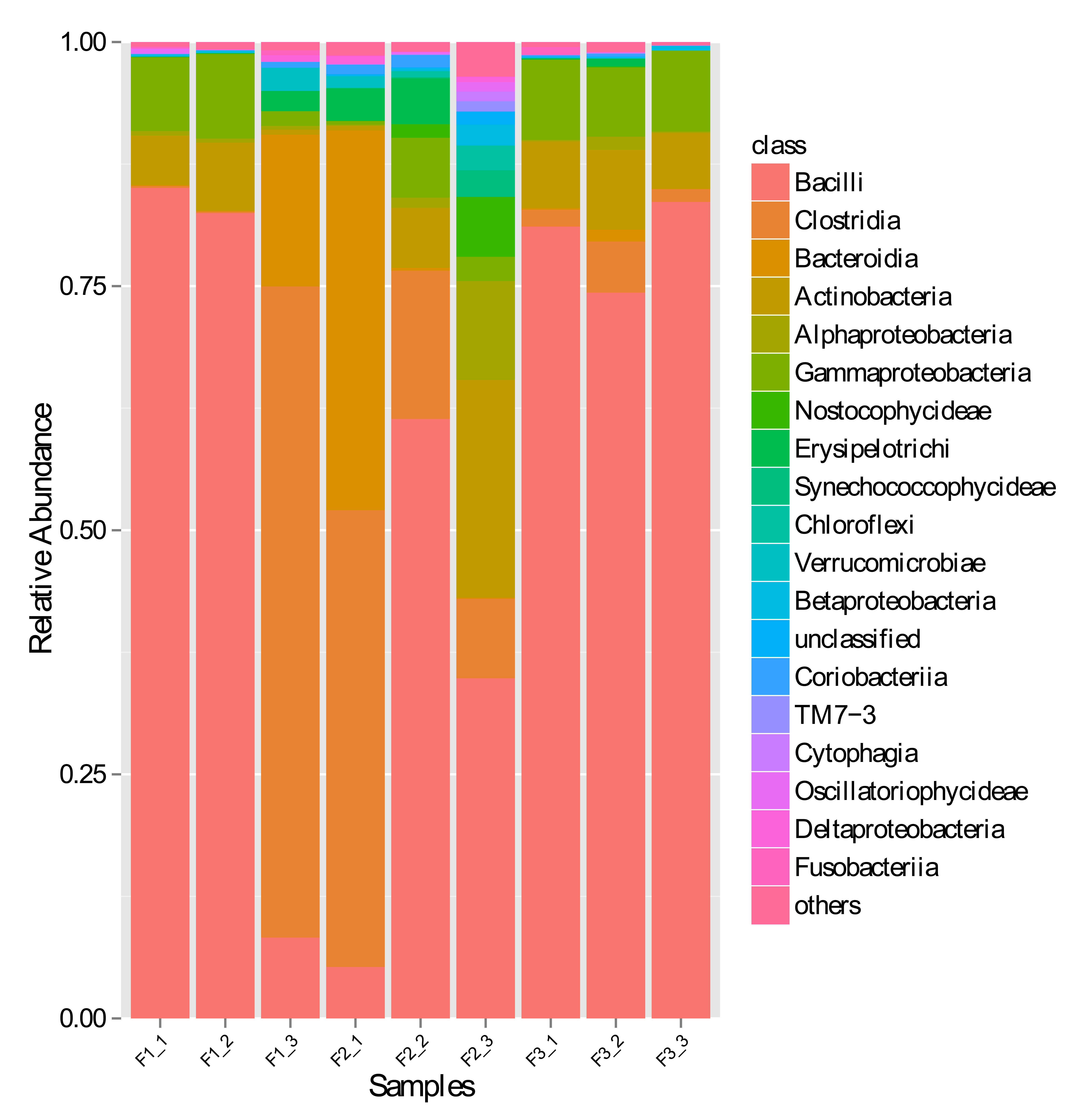

Supplement: Supplementary file 3 — Figure S3. Composition of Microbial Community at Class level. [file MBO3-5-287-s003.tif]

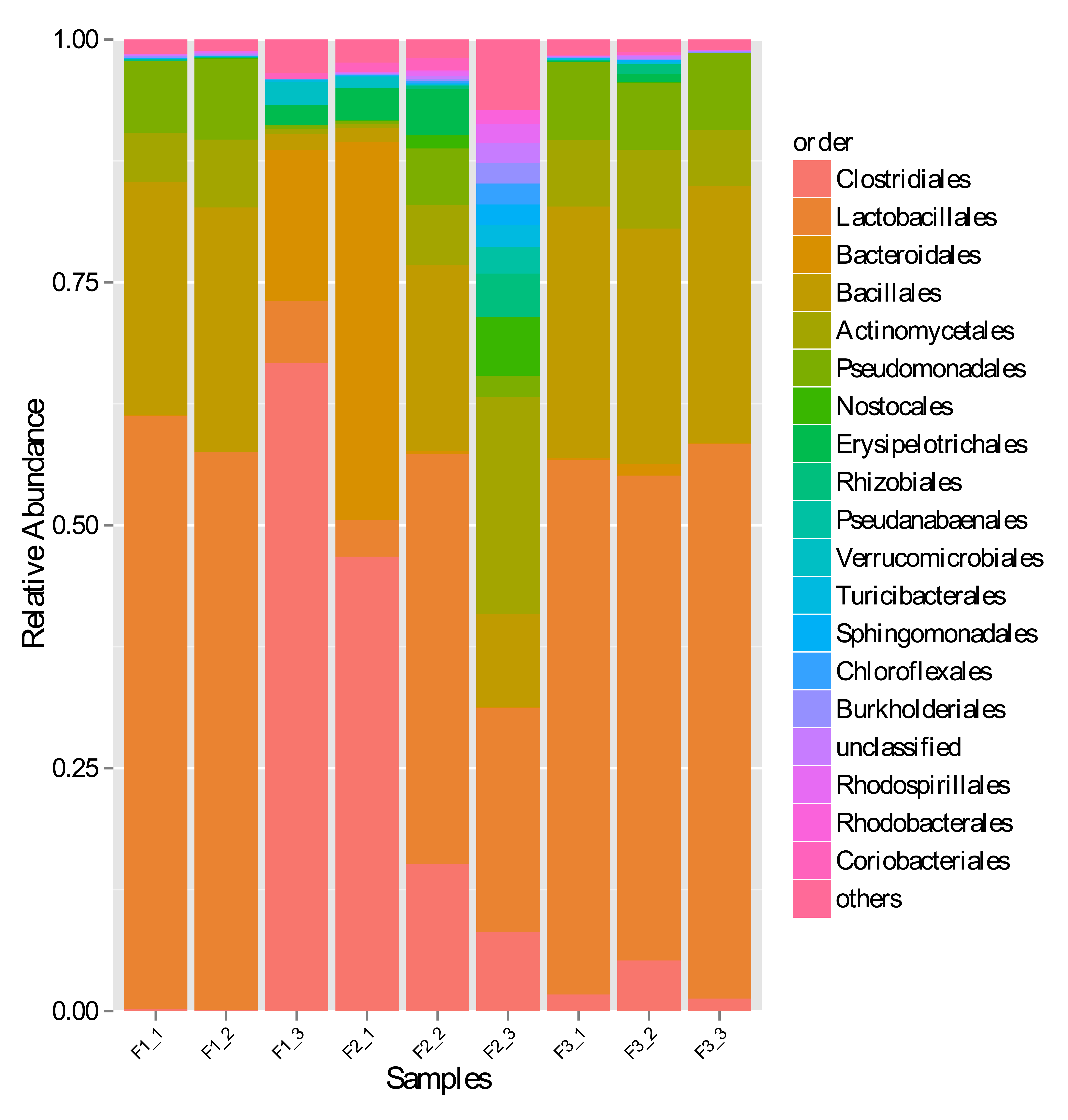

Supplement: Supplementary file 4 — Figure S4. Composition of Microbial Community at Order level. [file MBO3-5-287-s004.tif]

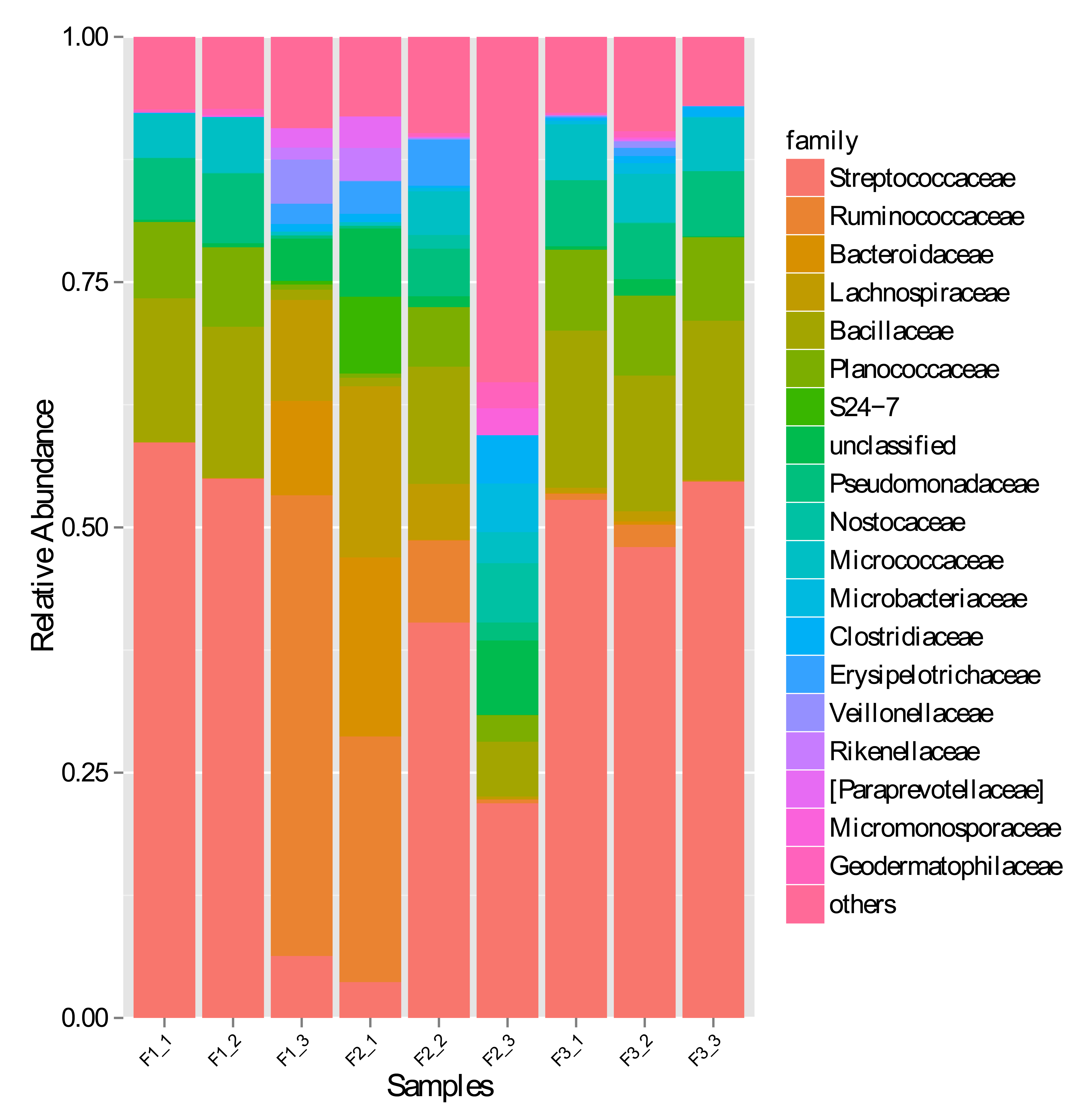

Supplement: Supplementary file 5 — Figure S5. Composition of Microbial Community at Family level. [file MBO3-5-287-s005.tif]
