# Supplementary material for: High‐throughput sequencing reveals the core gut microbiome of Bar‐headed goose (Anser indicus) in different wintering areas in Tibet
Source: Microbiologyopen. 2016 Feb 4;5(2):287–95. doi: 10.1002/mbo3.327 (PMC4831473; doi:10.1002/mbo3.327)
Supplement: Supplementary file 6 — Table S1. Raw data before and after standard quality control (QC) filters. [file MBO3-5-287-s006.docx]

**Table S1. Raw data before and after standard quality control (QC) filters.**

| Sample ID | Sequences | Base Pairs | Post QC Sequences | Post QC Base Pairs | Post QC  Mean Length |
| --- | --- | --- | --- | --- | --- |
| F1_1 | 62,276 | 18,745,076 | 24,850 | 11,146,652 | 448.56 |
| F1_2 | 80,100 | 24,110,100 | 34,340 | 15,403,750 | 448.57 |
| F1_3 | 55,548 | 16,719,948 | 21,167 | 9,170,293 | 433.24 |
| F2_1 | 61,198 | 18,420,598 | 23,439 | 10,218,602 | 435.97 |
| F2_2 | 72,678 | 21,876,078 | 31,205 | 13,851,917 | 443.90 |
| F2_3 | 72,282 | 21,756,882 | 30,681 | 13,385,972 | 436.30 |
| F3_1 | 57,378 | 17,270,778 | 22,675 | 10,161,413 | 448.13 |
| F3_2 | 60,230 | 18,129,230 | 23,301 | 10,371,672 | 445.12 |
| F3_3 | 64,178 | 19,317,578 | 25,018 | 11,224,992 | 448.68 |
