# Supplementary material for: High‐throughput sequencing reveals the core gut microbiome of Bar‐headed goose (Anser indicus) in different wintering areas in Tibet
Source: Microbiologyopen. 2016 Feb 4;5(2):287–95. doi: 10.1002/mbo3.327 (PMC4831473; doi:10.1002/mbo3.327)
Supplement: Supplementary file 7 — Table S2. Number of bacterial taxonomic units. [file MBO3-5-287-s007.docx]

**Table S2. Number of bacterial taxonomic units.**

| Sample ID | Number of taxonomic units | | | | |
| --- | --- | --- | --- | --- | --- |
|  | Phylum | Class | Order | Family | Genus |
| F1_1 | 9 | 27 | 37 | 67 | 73 |
| F1_2 | 11 | 34 | 42 | 80 | 83 |
| F1_3 | 11 | 24 | 30 | 62 | 70 |
| F2_1 | 11 | 27 | 34 | 65 | 69 |
| F2_2 | 12 | 38 | 53 | 97 | 117 |
| F2_3 | 13 | 39 | 53 | 103 | 113 |
| F3_1 | 10 | 26 | 34 | 75 | 82 |
| F3_2 | 9 | 26 | 38 | 82 | 93 |
| F3_3 | 7 | 17 | 25 | 57 | 67 |
| Total | 14 | 42 | 66 | 124 | 150 |
