# Supplementary material for: High‐throughput sequencing reveals the core gut microbiome of Bar‐headed goose (Anser indicus) in different wintering areas in Tibet
Source: Microbiologyopen. 2016 Feb 4;5(2):287–95. doi: 10.1002/mbo3.327 (PMC4831473; doi:10.1002/mbo3.327)
Supplement: Supplementary file 8 — Table S3. The taxonomic composition at the phylum level of the microbial communities in each sample. [file MBO3-5-287-s008.docx]

**Table S3. The taxonomic composition at the phylum level of the microbial communities in each sample.**

| Phylum | Relative Abundance (%) | | | | | | | | | |
| --- | --- | --- | --- | --- | --- | --- | --- | --- | --- | --- |
|  | F1_1 | F1_2 | F1_3 | F2_1 | F2_2 | F2_3 | F3_1 | F3_2 | F3_3 | Average |
| Firmicutes | 85.27 | 82.64 | 77.04 | 55.38 | 81.28 | 43.05 | 82.98 | 80.41 | 84.94 | 74.78 |
| Proteobacteria | 8.26 | 9.32 | 2.58 | 1.33 | 7.52 | 15.32 | 8.55 | 8.74 | 8.91 | 7.84 |
| Actinobacteria | 5.10 | 7.12 | 1.11 | 1.45 | 7.59 | 23.44 | 7.05 | 8.82 | 5.75 | 7.49 |
| Bacteroidetes | 0.44 | 0.34 | 15.56 | 39.09 | 0.64 | 1.60 | 0.42 | 1.43 | 0.30 | 6.65 |
| Cyanobacteria | 0.54 | 0.11 | 0.04 | 0.84 | 1.75 | 10.49 | 0.01 | 0.09 | 0.00 | 1.54 |
| Chloroflexi | 0.09 | 0.15 | 0.01 | 0.09 | 0.93 | 3.57 | 0.04 | 0.32 | 0.02 | 0.58 |
| Verrucomicrobia | 0.00 | 0.01 | 2.35 | 1.14 | 0.01 | 0.04 | 0.00 | 0.00 | 0.00 | 0.39 |
| TM7 | 0.04 | 0.04 | 0.01 | 0.03 | 0.09 | 1.72 | 0.04 | 0.11 | 0.06 | 0.24 |
| Tenericutes | 0.00 | 0.15 | 0.72 | 0.57 | 0.07 | 0.00 | 0.10 | 0.06 | 0.01 | 0.19 |
| Fusobacteria | 0.19 | 0.02 | 0.51 | 0.08 | 0.03 | 0.01 | 0.80 | 0.00 | 0.00 | 0.18 |
| Acidobacteria | 0.07 | 0.10 | 0.00 | 0.01 | 0.09 | 0.71 | 0.01 | 0.01 | 0.00 | 0.11 |
| Synergistetes | 0.00 | 0.00 | 0.07 | 0.00 | 0.00 | 0.00 | 0.00 | 0.00 | 0.00 | 0.01 |
| Planctomycetes | 0.00 | 0.00 | 0.00 | 0.00 | 0.00 | 0.01 | 0.00 | 0.00 | 0.00 | 0.00 |
| Gemmatimonadetes | 0.00 | 0.00 | 0.00 | 0.00 | 0.00 | 0.02 | 0.00 | 0.00 | 0.00 | 0.00 |
