# Supplementary material for: High‐throughput sequencing reveals the core gut microbiome of Bar‐headed goose (Anser indicus) in different wintering areas in Tibet
Source: Microbiologyopen. 2016 Feb 4;5(2):287–95. doi: 10.1002/mbo3.327 (PMC4831473; doi:10.1002/mbo3.327)
Supplement: Supplementary file 9 — Table S4. The distribution of the sequences belonged to different genera in each sample. [file MBO3-5-287-s009.docx]

**Table S4. The distribution of the sequences belonged to different genera in each sample.**

| **Genus** | **F1_1** | **F1_2** | **F1_3** | **F2_1** | **F2_2** | **F2_3** | **F3_1** | **F3_2** | **F3_3** |
| --- | --- | --- | --- | --- | --- | --- | --- | --- | --- |
| Slackia | 0 | 0 | 4 | 6 | 34 | 0 | 0 | 0 | 0 |
| Streptococcus | 457 | 651 | 337 | 23 | 453 | 175 | 393 | 302 | 547 |
| Vagococcus | 5 | 3 | 0 | 0 | 2 | 0 | 1 | 1 | 5 |
| Peptococcus | 0 | 0 | 133 | 120 | 166 | 1 | 6 | 24 | 0 |
| cc_115 | 0 | 0 | 1 | 26 | 29 | 0 | 0 | 0 | 0 |
| Haliangium | 0 | 0 | 0 | 0 | 1 | 9 | 0 | 0 | 0 |
| Roseomonas | 0 | 2 | 0 | 0 | 2 | 11 | 0 | 0 | 0 |
| Pedomicrobium | 0 | 0 | 0 | 0 | 0 | 5 | 0 | 4 | 0 |
| Solibacillus | 1463 | 2128 | 68 | 58 | 1416 | 432 | 1372 | 1091 | 1624 |
| Pseudomonas | 1442 | 2283 | 52 | 45 | 1409 | 384 | 1438 | 1097 | 1573 |
| Barnesiella | 0 | 0 | 21 | 230 | 1 | 0 | 0 | 0 | 0 |
| Kineococcus | 4 | 0 | 0 | 1 | 8 | 183 | 0 | 0 | 1 |
| Psychrobacter | 154 | 243 | 5 | 10 | 171 | 46 | 165 | 127 | 177 |
| [Eubacterium] | 0 | 0 | 23 | 84 | 481 | 0 | 0 | 1 | 0 |
| Virgisporangium | 2 | 24 | 0 | 0 | 3 | 38 | 0 | 7 | 1 |
| YRC22 | 0 | 1 | 85 | 9 | 3 | 0 | 3 | 8 | 0 |
| Kaistobacter | 15 | 13 | 0 | 1 | 14 | 280 | 1 | 33 | 0 |
| Thalassobacillus | 0 | 0 | 0 | 0 | 0 | 1 | 1 | 0 | 0 |
| Corynebacterium | 0 | 6 | 0 | 0 | 0 | 0 | 0 | 1 | 1 |
| Microbispora | 3 | 3 | 0 | 0 | 17 | 137 | 5 | 26 | 2 |
| Mycobacterium | 5 | 17 | 0 | 0 | 12 | 235 | 8 | 36 | 3 |
| Acinetobacter | 29 | 52 | 3 | 1 | 62 | 11 | 41 | 40 | 51 |
| Solirubrobacter | 0 | 8 | 0 | 1 | 12 | 54 | 6 | 14 | 1 |
| **Genus** | **F1_1** | **F1_2** | **F1_3** | **F2_1** | **F2_2** | **F2_3** | **F3_1** | **F3_2** | **F3_3** |
| Rhodoplanes | 0 | 1 | 0 | 0 | 3 | 28 | 0 | 12 | 0 |
| Enterococcus | 13 | 23 | 0 | 0 | 20 | 14 | 8 | 4 | 15 |
| Enhydrobacter | 66 | 102 | 3 | 4 | 53 | 19 | 68 | 56 | 78 |
| Ureaplasma | 0 | 43 | 0 | 0 | 0 | 0 | 0 | 0 | 0 |
| Lysobacter | 0 | 0 | 0 | 0 | 0 | 14 | 0 | 0 | 0 |
| Rathayibacter | 3 | 0 | 0 | 0 | 33 | 206 | 4 | 7 | 1 |
| Streptosporangium | 0 | 0 | 0 | 0 | 0 | 4 | 2 | 5 | 0 |
| Phormidium | 106 | 4 | 2 | 7 | 54 | 204 | 0 | 0 | 0 |
| Leuconostoc | 60 | 180 | 4 | 4 | 109 | 24 | 120 | 60 | 114 |
| Rubrobacter | 0 | 4 | 0 | 0 | 7 | 10 | 2 | 8 | 0 |
| Anaerostipes | 0 | 0 | 12 | 0 | 0 | 2 | 0 | 0 | 0 |
| Mycetocola | 0 | 1 | 0 | 0 | 12 | 92 | 18 | 42 | 4 |
| Promicromonospora | 1 | 0 | 1 | 0 | 0 | 8 | 3 | 4 | 1 |
| Amaricoccus | 0 | 0 | 0 | 0 | 2 | 12 | 0 | 1 | 0 |
| Clostridium | 8 | 0 | 13 | 103 | 21 | 81 | 17 | 53 | 6 |
| Nostoc | 3 | 2 | 0 | 0 | 7 | 26 | 0 | 0 | 0 |
| Lysinibacillus | 301 | 449 | 17 | 16 | 321 | 96 | 332 | 295 | 370 |
| Agrobacterium | 1 | 3 | 1 | 0 | 13 | 245 | 6 | 81 | 7 |
| Tepidimicrobium | 0 | 0 | 0 | 0 | 0 | 0 | 1 | 2 | 0 |
| Escherichia | 24 | 38 | 173 | 3 | 62 | 15 | 34 | 25 | 40 |
| Dehalobacterium | 0 | 0 | 0 | 5 | 0 | 0 | 0 | 0 | 0 |
| Asteroleplasma | 0 | 3 | 0 | 0 | 2 | 0 | 1 | 0 | 0 |
| Nodularia | 1 | 0 | 0 | 0 | 34 | 20 | 0 | 0 | 0 |
| Bulleidia | 0 | 0 | 0 | 0 | 19 | 0 | 0 | 11 | 0 |
| **Genus** | **F1_1** | **F1_2** | **F1_3** | **F2_1** | **F2_2** | **F2_3** | **F3_1** | **F3_2** | **F3_3** |
| Chloronema | 0 | 2 | 0 | 9 | 42 | 164 | 0 | 0 | 0 |
| Polaromonas | 5 | 2 | 2 | 0 | 9 | 39 | 1 | 0 | 0 |
| Arcobacter | 1 | 1 | 2 | 0 | 2 | 1 | 1 | 0 | 3 |
| Megamonas | 0 | 2 | 597 | 2 | 13 | 1 | 28 | 129 | 0 |
| Exiguobacterium | 0 | 0 | 0 | 0 | 2 | 41 | 21 | 12 | 4 |
| Actinomadura | 0 | 9 | 1 | 0 | 0 | 0 | 0 | 0 | 0 |
| Stenotrophomonas | 1 | 0 | 0 | 0 | 3 | 4 | 0 | 1 | 5 |
| Adhaeribacter | 0 | 0 | 0 | 0 | 9 | 46 | 0 | 0 | 0 |
| Coprococcus | 0 | 0 | 111 | 373 | 15 | 10 | 10 | 2 | 3 |
| Balneimonas | 8 | 16 | 2 | 3 | 36 | 163 | 5 | 24 | 0 |
| Fusobacterium | 43 | 5 | 0 | 0 | 0 | 0 | 0 | 0 | 0 |
| Skermanella | 21 | 41 | 1 | 4 | 131 | 327 | 0 | 10 | 5 |
| Bradyrhizobium | 6 | 4 | 0 | 0 | 4 | 68 | 1 | 34 | 0 |
| Helicobacter | 3 | 0 | 0 | 0 | 0 | 2 | 3 | 1 | 0 |
| Brochothrix | 104 | 178 | 2 | 2 | 108 | 29 | 90 | 87 | 103 |
| Cupriavidus | 2 | 0 | 0 | 1 | 2 | 1 | 1 | 1 | 1 |
| Agromyces | 0 | 1 | 0 | 0 | 1 | 21 | 1 | 4 | 0 |
| Bacillus | 3374 | 4945 | 172 | 153 | 3463 | 1161 | 3419 | 2649 | 3854 |
| Collinsella | 0 | 0 | 1 | 23 | 37 | 0 | 0 | 0 | 0 |
| Pseudonocardia | 18 | 52 | 2 | 1 | 48 | 96 | 7 | 14 | 1 |
| Anabaena | 0 | 0 | 0 | 0 | 0 | 0 | 0 | 3 | 0 |
| SMB53 | 10 | 12 | 114 | 43 | 41 | 868 | 44 | 86 | 242 |
| Blautia | 0 | 1 | 40 | 174 | 91 | 1 | 2 | 1 | 0 |
| Acetobacter | 3 | 1 | 0 | 0 | 2 | 1 | 2 | 2 | 5 |
| **Genus** | **F1_1** | **F1_2** | **F1_3** | **F2_1** | **F2_2** | **F2_3** | **F3_1** | **F3_2** | **F3_3** |
| Rhodobacter | 1 | 2 | 0 | 0 | 2 | 21 | 0 | 2 | 0 |
| Carnobacterium | 259 | 463 | 8 | 12 | 316 | 111 | 256 | 199 | 329 |
| Arthrobacter | 1006 | 1794 | 60 | 59 | 1250 | 365 | 1186 | 902 | 1295 |
| Coprobacillus | 0 | 0 | 4 | 71 | 49 | 0 | 0 | 2 | 0 |
| Flavobacterium | 29 | 46 | 2 | 2 | 36 | 24 | 25 | 13 | 31 |
| Nannocystis | 0 | 0 | 0 | 0 | 0 | 2 | 0 | 6 | 0 |
| Ramlibacter | 2 | 1 | 0 | 0 | 4 | 28 | 0 | 0 | 0 |
| Azospirillum | 1 | 0 | 0 | 0 | 2 | 2 | 0 | 0 | 0 |
| Chryseobacterium | 7 | 16 | 0 | 1 | 6 | 2 | 6 | 8 | 9 |
| Perlucidibaca | 0 | 0 | 0 | 0 | 1 | 0 | 0 | 2 | 1 |
| Phascolarctobacterium | 0 | 1 | 141 | 12 | 15 | 0 | 0 | 0 | 0 |
| Sphingomonas | 16 | 9 | 0 | 2 | 7 | 105 | 0 | 2 | 1 |
| Wautersiella | 2 | 7 | 0 | 0 | 8 | 0 | 7 | 5 | 3 |
| Veillonella | 0 | 0 | 0 | 0 | 7 | 0 | 0 | 0 | 11 |
| Rothia | 18 | 13 | 1 | 0 | 4 | 42 | 1 | 3 | 2 |
| Paenibacillus | 3 | 0 | 1 | 0 | 1 | 16 | 0 | 0 | 0 |
| Rubellimicrobium | 4 | 1 | 3 | 1 | 38 | 252 | 0 | 3 | 4 |
| Saccharomonospora | 0 | 0 | 0 | 0 | 0 | 4 | 3 | 1 | 0 |
| Akkermansia | 0 | 3 | 386 | 200 | 1 | 1 | 0 | 0 | 0 |
| Salinibacterium | 2 | 4 | 0 | 4 | 40 | 424 | 48 | 124 | 13 |
| Sporosarcina | 21 | 27 | 1 | 0 | 23 | 18 | 10 | 25 | 25 |
| Tepidibacter | 0 | 0 | 0 | 0 | 3 | 29 | 4 | 13 | 0 |
| Methylobacterium | 3 | 3 | 1 | 1 | 1 | 23 | 1 | 0 | 1 |
| Nonomuraea | 0 | 3 | 0 | 0 | 0 | 7 | 0 | 0 | 0 |
| **Genus** | **F1_1** | **F1_2** | **F1_3** | **F2_1** | **F2_2** | **F2_3** | **F3_1** | **F3_2** | **F3_3** |
| Oscillospira | 0 | 1 | 765 | 433 | 60 | 5 | 7 | 5 | 0 |
| Cylindrospermopsis | 0 | 0 | 0 | 0 | 5 | 0 | 0 | 0 | 0 |
| Turicibacter | 6 | 0 | 39 | 16 | 19 | 462 | 38 | 41 | 6 |
| Hymenobacter | 3 | 0 | 0 | 1 | 3 | 51 | 0 | 0 | 0 |
| Sanguibacter | 0 | 4 | 1 | 0 | 15 | 63 | 2 | 1 | 0 |
| Neisseria | 0 | 0 | 0 | 0 | 0 | 0 | 0 | 0 | 79 |
| Actinoplanes | 1 | 3 | 2 | 0 | 2 | 208 | 2 | 3 | 0 |
| Planomicrobium | 1 | 1 | 0 | 0 | 2 | 22 | 47 | 158 | 4 |
| Janthinobacterium | 23 | 25 | 1 | 0 | 9 | 21 | 17 | 16 | 15 |
| Parvimonas | 7 | 0 | 0 | 0 | 0 | 0 | 0 | 0 | 0 |
| Cryptosporangium | 0 | 1 | 1 | 0 | 0 | 20 | 0 | 0 | 0 |
| Serratia | 7 | 28 | 1 | 0 | 7 | 10 | 7 | 16 | 40 |
| Peptoniphilus | 5 | 0 | 0 | 0 | 0 | 0 | 2 | 0 | 1 |
| Butyricicoccus | 0 | 3 | 382 | 353 | 140 | 0 | 2 | 8 | 0 |
| gut | 0 | 0 | 1 | 4 | 1 | 0 | 0 | 0 | 0 |
| Campylobacter | 0 | 0 | 0 | 0 | 6 | 3 | 0 | 1 | 0 |
| Myroides | 24 | 14 | 0 | 0 | 32 | 7 | 16 | 17 | 21 |
| Nocardioides | 0 | 3 | 0 | 0 | 1 | 17 | 1 | 1 | 0 |
| Prevotella | 0 | 2 | 339 | 103 | 1 | 0 | 1 | 108 | 0 |
| Rhodocytophaga | 4 | 0 | 0 | 1 | 1 | 68 | 0 | 0 | 0 |
| Propionibacterium | 0 | 13 | 0 | 0 | 12 | 2 | 3 | 4 | 14 |
| Bacteroides | 2 | 6 | 1578 | 3190 | 18 | 4 | 18 | 63 | 1 |
| Rikenella | 0 | 0 | 1 | 108 | 5 | 0 | 0 | 0 | 0 |
| Rhodococcus | 4 | 0 | 0 | 2 | 15 | 338 | 33 | 16 | 1 |
| **Genus** | **F1_1** | **F1_2** | **F1_3** | **F2_1** | **F2_2** | **F2_3** | **F3_1** | **F3_2** | **F3_3** |
| Parabacteroides | 0 | 0 | 0 | 169 | 21 | 0 | 0 | 0 | 0 |
| Williamsia | 0 | 0 | 0 | 0 | 0 | 18 | 0 | 0 | 0 |
| Anaerofilum | 0 | 0 | 8 | 15 | 0 | 0 | 0 | 0 | 0 |
| Alkalibacillus | 0 | 3 | 0 | 0 | 6 | 0 | 0 | 0 | 0 |
| Paracoccus | 0 | 2 | 1 | 0 | 4 | 9 | 2 | 0 | 0 |
| Leptolyngbya | 5 | 6 | 1 | 4 | 24 | 325 | 0 | 0 | 0 |
| Flavisolibacter | 0 | 0 | 0 | 0 | 0 | 19 | 0 | 0 | 0 |
| Oscillochloris | 0 | 0 | 0 | 0 | 3 | 11 | 0 | 0 | 0 |
| Anaerofustis | 0 | 0 | 0 | 9 | 7 | 0 | 0 | 0 | 0 |
| Planifilum | 0 | 0 | 0 | 0 | 0 | 9 | 2 | 6 | 0 |
| Paraprevotella | 0 | 0 | 16 | 19 | 0 | 0 | 0 | 0 | 0 |
| Helcococcus | 7 | 0 | 0 | 0 | 1 | 1 | 0 | 0 | 2 |
| Ruminococcus | 0 | 0 | 192 | 116 | 35 | 34 | 0 | 0 | 1 |
| Faecalibacterium | 0 | 0 | 1266 | 292 | 27 | 4 | 6 | 8 | 0 |
| Mogibacterium | 0 | 0 | 0 | 0 | 1 | 33 | 4 | 0 | 3 |
| Lactococcus | 13023 | 17014 | 700 | 616 | 11253 | 4418 | 10890 | 8940 | 12450 |
| Phenylobacterium | 0 | 2 | 0 | 0 | 0 | 4 | 0 | 0 | 0 |
| [Ruminococcus] | 0 | 1 | 492 | 1248 | 306 | 1 | 61 | 114 | 32 |
| Butyricimonas | 0 | 0 | 8 | 2 | 0 | 0 | 0 | 0 | 2 |
| [Prevotella] | 0 | 0 | 0 | 1 | 16 | 0 | 0 | 13 | 0 |
| Devosia | 2 | 0 | 1 | 0 | 6 | 133 | 1 | 21 | 0 |
| Porphyromonas | 23 | 10 | 0 | 0 | 4 | 2 | 7 | 1 | 4 |
| Cellulomonas | 3 | 2 | 0 | 0 | 16 | 91 | 2 | 17 | 1 |
| Dyadobacter | 5 | 1 | 0 | 0 | 0 | 12 | 0 | 0 | 0 |
| **Genus** | **F1_1** | **F1_2** | **F1_3** | **F2_1** | **F2_2** | **F2_3** | **F3_1** | **F3_2** | **F3_3** |
| Legionella | 0 | 0 | 0 | 0 | 0 | 3 | 0 | 0 | 0 |
| Lactobacillus | 211 | 105 | 2 | 3 | 91 | 99 | 79 | 99 | 114 |
| Hyphomicrobium | 0 | 0 | 0 | 1 | 0 | 12 | 0 | 2 | 0 |
| Kineosporia | 2 | 4 | 1 | 0 | 4 | 67 | 0 | 1 | 1 |
| Alicyclobacillus | 0 | 0 | 0 | 0 | 0 | 3 | 1 | 14 | 2 |
| Salana | 0 | 0 | 0 | 0 | 0 | 8 | 2 | 0 | 0 |
| Desulfovibrio | 0 | 0 | 110 | 140 | 4 | 0 | 0 | 2 | 0 |
| unclassified | 475 | 940 | 7888 | 8723 | 5921 | 6733 | 874 | 1712 | 405 |
