# Supplementary material for: High‐throughput sequencing reveals the core gut microbiome of Bar‐headed goose (Anser indicus) in different wintering areas in Tibet
Source: Microbiologyopen. 2016 Feb 4;5(2):287–95. doi: 10.1002/mbo3.327 (PMC4831473; doi:10.1002/mbo3.327)
Supplement: Supplementary file 10 — Table S5. The top 18 most abundant genera in each sample. [file MBO3-5-287-s010.docx]

**Table S5. The top 18 most abundant genera in each sample.**

| Genus | Relative Abundance (%) | | | | | | | | | |
| --- | --- | --- | --- | --- | --- | --- | --- | --- | --- | --- |
|  | F1_1 | F1_2 | F1_3 | F2_1 | F2_2 | F2_3 | F3_1 | F3_2 | F3_3 | Average |
| Lactococcus | 56.67 | 52.93 | 4.27 | 3.52 | 38.74 | 21.05 | 50.97 | 46.43 | 52.37 | 36.33 |
| unclassified | 2.07 | 2.92 | 48.07 | 49.91 | 20.39 | 32.07 | 4.09 | 8.89 | 1.70 | 18.90 |
| Bacillus | 14.68 | 15.38 | 1.05 | 0.88 | 11.92 | 5.53 | 16.00 | 13.76 | 16.21 | 10.60 |
| Pseudomonas | 6.27 | 7.10 | 0.32 | 0.26 | 4.85 | 1.83 | 6.73 | 5.70 | 6.62 | 4.41 |
| Solibacillus | 6.37 | 6.62 | 0.41 | 0.33 | 4.88 | 2.06 | 6.42 | 5.67 | 6.83 | 4.40 |
| Arthrobacter | 4.38 | 5.58 | 0.37 | 0.34 | 4.30 | 1.74 | 5.55 | 4.68 | 5.45 | 3.60 |
| Bacteroides | 0.01 | 0.02 | 9.62 | 18.25 | 0.06 | 0.02 | 0.08 | 0.33 | 0.00 | 3.15 |
| Streptococcus | 1.99 | 2.03 | 2.05 | 0.13 | 1.56 | 0.83 | 1.84 | 1.57 | 2.30 | 1.59 |
| [Ruminococcus] | 0.00 | 0.00 | 3.00 | 7.14 | 1.05 | 0.00 | 0.29 | 0.59 | 0.13 | 1.36 |
| Faecalibacterium | 0.00 | 0.00 | 7.71 | 1.67 | 0.09 | 0.02 | 0.03 | 0.04 | 0.00 | 1.06 |
| Oscillospira | 0.00 | 0.00 | 4.66 | 2.48 | 0.21 | 0.02 | 0.03 | 0.03 | 0.00 | 0.83 |
| SMB53 | 0.04 | 0.04 | 0.69 | 0.25 | 0.14 | 4.13 | 0.21 | 0.45 | 1.02 | 0.77 |
| Butyricicoccus | 0.00 | 0.01 | 2.33 | 2.02 | 0.48 | 0.00 | 0.01 | 0.04 | 0.00 | 0.54 |
| Megamonas | 0.00 | 0.01 | 3.64 | 0.01 | 0.04 | 0.00 | 0.13 | 0.67 | 0.00 | 0.50 |
| Akkermansia | 0.00 | 0.01 | 2.35 | 1.14 | 0.00 | 0.00 | 0.00 | 0.00 | 0.00 | 0.39 |
| Prevotella | 0.00 | 0.01 | 2.07 | 0.59 | 0.00 | 0.00 | 0.00 | 0.56 | 0.00 | 0.36 |
| Salinibacterium | 0.01 | 0.01 | 0.00 | 0.02 | 0.14 | 2.02 | 0.22 | 0.64 | 0.05 | 0.35 |
| Turicibacter | 0.03 | 0.00 | 0.24 | 0.09 | 0.07 | 2.20 | 0.18 | 0.21 | 0.03 | 0.34 |
| Coprococcus | 0.00 | 0.00 | 0.68 | 2.13 | 0.05 | 0.05 | 0.05 | 0.01 | 0.01 | 0.33 |
