# Supplementary material for: High‐throughput sequencing reveals the core gut microbiome of Bar‐headed goose (Anser indicus) in different wintering areas in Tibet
Source: Microbiologyopen. 2016 Feb 4;5(2):287–95. doi: 10.1002/mbo3.327 (PMC4831473; doi:10.1002/mbo3.327)
Supplement: Supplementary file 11 — Table S6. The distribution of sequences belonged to the newly added genera at 88.9 and 77.9% core threshold. [file MBO3-5-287-s011.docx]

**Table S6. The distribution of sequences belonged to the newly added genera at 88.9% and 77.9% core threshold.**

|  | Genus | F1_1 | F1_2 | F1_3 | F2_1 | F2_2 | F2_3 | F3_1 | F3_2 | F3_3 |
| --- | --- | --- | --- | --- | --- | --- | --- | --- | --- | --- |
| 88.9% core threshold | [Ruminococcus] | 0 | 1 | 492 | 1248 | 306 | 1 | 61 | 114 | 32 |
|  | Salinibacterium | 2 | 4 | 0 | 4 | 40 | 424 | 48 | 124 | 13 |
|  | Turicibacter | 6 | 0 | 39 | 16 | 19 | 462 | 38 | 41 | 6 |
|  | Skermanella | 21 | 41 | 1 | 4 | 131 | 327 | 0 | 10 | 5 |
|  | Agrobacterium | 1 | 3 | 1 | 0 | 13 | 245 | 6 | 81 | 7 |
|  | Rubellimicrobium | 4 | 1 | 3 | 1 | 38 | 252 | 0 | 3 | 4 |
|  | Clostridium | 8 | 0 | 13 | 103 | 21 | 81 | 17 | 53 | 6 |
|  | Balneimonas | 8 | 16 | 2 | 3 | 36 | 163 | 5 | 24 | 0 |
|  | Sporosarcina | 21 | 27 | 1 | 0 | 23 | 18 | 10 | 25 | 25 |
|  | Janthinobacterium | 23 | 25 | 1 | 0 | 9 | 21 | 17 | 16 | 15 |
|  | Serratia | 7 | 28 | 1 | 0 | 7 | 10 | 7 | 16 | 40 |
|  | Rothia | 18 | 13 | 1 | 0 | 4 | 42 | 1 | 3 | 2 |
|  | Chryseobacterium | 7 | 16 | 0 | 1 | 6 | 2 | 6 | 8 | 9 |
|  | Methylobacterium | 3 | 3 | 1 | 1 | 1 | 23 | 1 | 0 | 1 |
| 77.9% core threshold | Oscillospira | 0 | 1 | 765 | 433 | 60 | 5 | 7 | 5 | 0 |
|  | Megamonas | 0 | 2 | 597 | 2 | 13 | 1 | 28 | 129 | 0 |
|  | Coprococcus | 0 | 0 | 111 | 373 | 15 | 10 | 10 | 2 | 3 |
|  | Kaistobacter | 15 | 13 | 0 | 1 | 14 | 280 | 1 | 33 | 0 |
|  | Mycobacterium | 5 | 17 | 0 | 0 | 12 | 235 | 8 | 36 | 3 |
|  | Blautia | 0 | 1 | 40 | 174 | 91 | 1 | 2 | 1 | 0 |
|  | Planomicrobium | 1 | 1 | 0 | 0 | 2 | 22 | 47 | 158 | 4 |
|  | Actinoplanes | 1 | 3 | 2 | 0 | 2 | 208 | 2 | 3 | 0 |
|  | Microbispora | 3 | 3 | 0 | 0 | 17 | 137 | 5 | 26 | 2 |
|  | Sphingomonas | 16 | 9 | 0 | 2 | 7 | 105 | 0 | 2 | 1 |
|  | Cellulomonas | 3 | 2 | 0 | 0 | 16 | 91 | 2 | 17 | 1 |
|  | Enterococcus | 13 | 23 | 0 | 0 | 20 | 14 | 8 | 4 | 15 |
|  | Solirubrobacter | 0 | 8 | 0 | 1 | 12 | 54 | 6 | 14 | 1 |
|  | Kineosporia | 2 | 4 | 1 | 0 | 4 | 67 | 0 | 1 | 1 |
|  | Porphyromonas | 23 | 10 | 0 | 0 | 4 | 2 | 7 | 1 | 4 |
|  | Acetobacter | 3 | 1 | 0 | 0 | 2 | 1 | 2 | 2 | 5 |
|  | Arcobacter | 1 | 1 | 2 | 0 | 2 | 1 | 1 | 0 | 3 |
|  | Cupriavidus | 2 | 0 | 0 | 1 | 2 | 1 | 1 | 1 | 1 |
